# 732. Increase in the Proportion of Hospitalizations in the Setting of Methicillin-Susceptible vs. Resistant *Staphylococcus aureus* in New York City (NYC)

**DOI:** 10.1093/ofid/ofad500.793

**Published:** 2023-11-27

**Authors:** Maria Bromberg, Chaorui C Huang, John T Doucette, Deena Altman

**Affiliations:** Mount Sinai School of Medicine, Brooklyn, New York; New York City Department of Health and Mental Hygiene, New York; Icahn School of Medicine at Mount Sinai, New York, New York; Icahn School of Medicine at Mount Sinai, New York, New York

## Abstract

**Background:**

Methicillin-resistant *Staphylococcus aureus* (MRSA) and methicillin-susceptible SA (MSSA) infections are a public health threat; MSSA is a growing problem requiring targeted attention. Socioeconomically disadvantaged patients face disparate outcomes. We analyzed SA infection trends in NYC.

**Methods:**

Hospitalization data for NYC residents were obtained from the NY SPARCS database for 2009-19. Trends in hospitalization rate by resistance (MSSA vs MRSA), clinical syndrome (bacteremia (BAC), pneumonia (PNA), all other infections (OTH)) and setting (community-associated (CA) vs healthcare-associated (HA)) were assessed with Poisson regression. Associations between baseline variables and death were assessed with multivariable logistic regression. CA SA cases were defined as those: 1) with no prior hospitalization in the past year; 2) with a SA diagnosis present on admission; 3) not admitted for complications from a surgery; 4) with no comorbid chronic conditions.

**Results:**

The overall rate of hospitalization with SA infection declined by 7.8% from 2009 to 2019. The hospitalization rate for all MSSA infections increased significantly (+21.8% BAC, +65.5% PNA, +18.1% OTH MSSA; p < 0.001 all groups) (F1). The rate of MRSA PNA increased significantly (+11%; p < 0.001) but MRSA BAC and OTH MRSA hospitalizations significantly declined (-25% and -30.4%, respectively; p < 0.001) (F1). CA cases declined as a proportion of all SA hospitalizations, from 18.4% in 2009 to 12.4% in 2019 (F2).

Age, living in a high-poverty area (HPA), and Medicaid or Medicare insurance were significantly associated with higher odds of in-hospital death for CA cases. Non-Hispanic Black and Hispanic Black patients had lower odds of death for HA but not CA cases; the effect of living in a HPA appeared to attenuate the association between race/ethnicity and death. A logistic regression model with demographic predictors (age, sex, race/ethnicity, HPA, insurance, and borough) was discriminative in predicting in-hospital death for CA cases (area under the curve: 0.87).

Crude Hospitalization Rates

**Figure:**
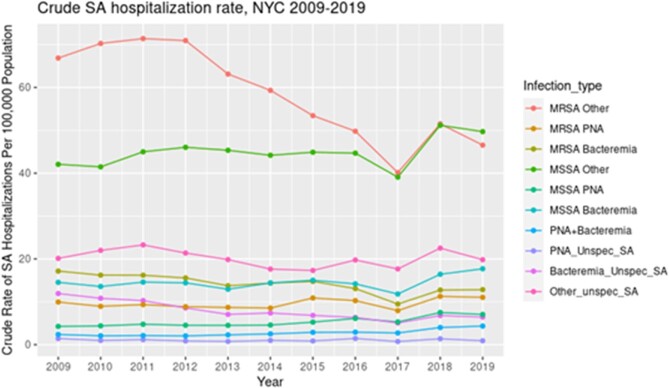

Crude Hospitalization Rates by Pathogen (MSSA, MRSA, and Unspecified SA infection) and Clinical Syndrome (Pneumonia, Bacteremia, and all other infections)

**Figure:**
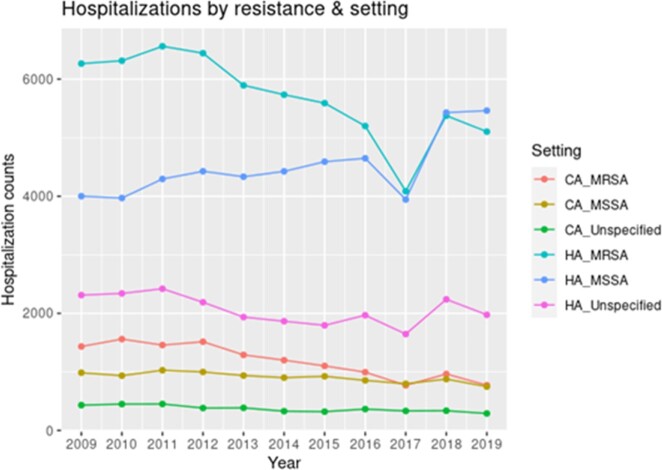

Total counts of hospitalizations by resistance (methicillin-resistant, methicillin-susceptionble, or unknown) and setting (hospital-associated or community-associated)

**Conclusion:**

While hospitalization with SA is declining overall, the relative burden of MSSA has increased compared to MRSA. Further studies and interventions are needed to understand this and baseline risk factors for SA mortality in vulnerable populations.

**Figure:**
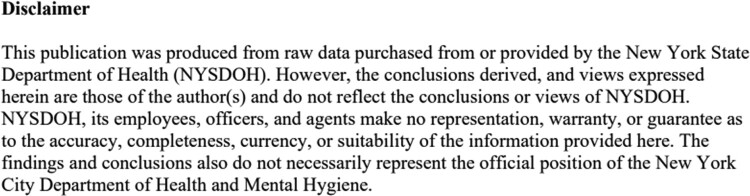

Mandatory disclaimer for publications using NYS Department of Health data and/or produced under auspices of NYC Department of Health and Mental Hygeine

**Disclosures:**

**All Authors**: No reported disclosures

